# Vascular endothelial growth factor A (VEGF-A) mRNA expression levels decrease after menopause in normal breast tissue but not in breast cancer lesions

**DOI:** 10.1038/sj.bjc.6690681

**Published:** 1999-09

**Authors:** R R Greb, I Maier, D Wallwiener, L Kiesel

**Affiliations:** Department of Obstetrics and Gynecology, University Hospital, Schleichstr. 4, Tübingen, D-72076, Germany

**Keywords:** breast cancer, angiogenesis, VEGF, steroid hormones, menopause

## Abstract

We hypothesized that the regulation of microvascular functions and angiogenesis in breast tissue, a well known target of ovarian steroid action, is dependent on the hormonal exposure of the breast. Relative expression levels of VEGF-A (vascular endothelial growth factor A), a putative key regulator of angiogenesis in breast cancer, were analysed in the tumour and the adjacent non-neoplastic breast tissue of 19 breast cancer patients by quantitative reverse transcriptase polymerase chain reaction. In non-neoplastic breast specimens the expression levels of all detected VEGF-A-isoforms (189, 165, 121) were significantly higher in premenopausal compared to post-menopausal women (*P* = 0.02) and were inversely correlated with the patient's age (*P* = 0.006). In contrast, in cancerous tissues menopausal status had no influence on VEGF-A-expression levels. Benign and malignant tissues exhibited a similar expression pattern of VEGF-A-isoforms relative to each other. Thus, the regulation of the vasculature in normal breast tissue, as opposed to breast cancer tissue, appears to be hormonally dependent. Endogenous and therapeutically used hormonal steroids might, therefore, cause clinically relevant changes of the angiogenic phenotype of the human breast. © 1999 Cancer Research Campaign


*British Journal of Cancer* (1999) **81**(2), 225–231
© 1999 Cancer Research Campaign
Article no. bjoc.1999.0681

### Vascular endothelial growth factor A (VEGF-A) mRNA expression levels decrease after menopause in normal breast tissue but not in breast cancer lesions

**RR Greb, I Maier, D Wallwiener and L Kiesel**

Department of Obstetrics and Gynecology, University Hospital, Schleichstr. 4, D-72076 Tübingen, Germany

**Summary** We hypothesized that the regulation of microvascular functions and angiogenesis in breast tissue, a well known target of ovarian steroid action, is dependent on the hormonal exposure of the breast. Relative expression levels of VEGF-A (vascular endothelial growth factor A), a putative key regulator of angiogenesis in breast cancer, were analysed in the tumour and the adjacent non-neoplastic breast tissue of 19 breast cancer patients by quantitative reverse transcriptase polymerase chain reaction. In non-neoplastic breast specimens the expression levels of all detected VEGF-A-isoforms (189, 165, 121) were significantly higher in premenopausal compared to post-menopausal women ($P = 0.02$) and were inversely correlated with the patient's age ($P = 0.006$). In contrast, in cancerous tissues menopausal status had no influence on VEGF-A-expression levels. Benign and malignant tissues exhibited a similar expression pattern of VEGF-A-isoforms relative to each other. Thus, the regulation of the vasculature in normal breast tissue, as opposed to breast cancer tissue, appears to be hormonally dependent. Endogenous and therapeutically used hormonal steroids might, therefore, cause clinically relevant changes of the angiogenic phenotype of the human breast.

© 1999 Cancer Research Campaign

**Keywords:** breast cancer; angiogenesis; VEGF; steroid hormones; menopause

The non-lactating female breast is subject to cyclic exposure of the ovarian hormones oestradiol and progesterone. In fact, breast tissue is highly responsive to changes in sex hormone concentrations and discrete histological stages can readily be identified throughout each menstrual cycle by expert breast pathologists (Vogel et al, 1981). Breast cancer cells frequently maintain hormone dependency, which was recognized at the end of the last century based upon the observation that metastatic breast cancer may remit after extirpation of the premenopausal woman's ovaries (Beatson, 1896). In today's clinical management of breast cancer, endocrine therapies, especially strategies antagonizing the action of oestradiol, are established adjuvant and palliative treatment options. In breast cancer, as well as in many other cancers, angiogenesis was identified as a crucial process for growth and metastasis since tumours must actively recruit blood supply in order to be able to grow beyond a size of 1–2 $mm^3$ (Gimbrone et al, 1972). Furthermore, evidence seen in animal models and humans link the onset of growth of dormant micrometastases to their acquisition of angiogenic activity (Hanahan and Folkman, 1996). Therefore, elucidating the mechanisms of angiogenesis is of enormous clinical importance to understand the mechanisms of disease progression, and to design preventive strategies. One of the most important and specific mediators of angiogenesis identified so far is vascular endothelial growth factor (VEGF). The initially characterized molecule is now termed VEGF-A and belongs to a family of structurally related peptides (VEGF-A, VEGF-B, VEGF-C, VEGF-D, PIGF). Different isoforms of VEGF-A arise by alternative splicing of mRNA and are named by the number of amino acids in the monomeric protein (VEGF189, VEGF165, VEGF121) (reviewed by Ferrara and Davis-Smyth, 1997). VEGF-A is strongly expressed in many human breast cancers and expression levels are closely correlated with microvessel density in angiogenic 'hot spots' (Toi et al, 1995). Cytosolic levels of VEGF in breast cancer tissue was identified as a prognostic marker in node-negative breast cancer patients with respect to relapse free and overall survival (Gasparini et al, 1997). Up to now, very little is known about the hormonal dependency of microvascular changes in normal breast and breast cancer. To some extent, angiogenesis in the human breast has been assessed functionally by Doppler sonography and magnetic resonance imaging (MRI). So far, these studies indicate a possible influence of the ovarian hormones on the vasculature. The assessment of tissue vascularization by Doppler sonographic measurements of vascular resistance and blood flow velocities suggests differences in vascular patterns between premenopausal and post-menopausal patients, and between tumour and normal breast tissue (Villena-Heinsen et al, 1998). Furthermore, parenchymal contrast enhancement patterns in MRI, which are thought to be caused by increased vessel density and permeability (Buadu et al, 1996), are changing depending on the stage of the patients' menstrual cycle (Müller-Schimpfle et al, 1997). Based upon these observations we hypothesized that the regulation of microvascular functions and angiogenesis in breast tissue, a well known target of ovarian steroid action, is likewise dependent on the hormonal milieu. VEGF-A, which has been demonstrated as a crucially important regulator of angiogenesis, seems to be a prime candidate to mediate hormonally induced changes on blood vessels in the breast. Preliminary evidence from studies in vitro suggests that oestrogens and progestins regulate the expression of VEGF in

*Received 13 January 1999*
*Revised 6 April 1999*
*Accepted 14 April 1999*
*Correspondence to:* RR Greb

**Table 1** Summary of VEGF-expression levels in breast carcinomas and the adjacent non-neoplastic tissues

| ID | Age yrs | MP | FSH IU/l | E2 pg/ml | P4 ng/ml | Histological type | Grade | TU size cm | LN metastasis | ER | PR | NNT VEGF-isoform | | | Ca VEGF-isoform | | |
|---|---|---|---|---|---|---|---|---|---|---|---|---|---|---|---|---|---|
| | | | | | | | | | | | | 189 | 165 | 121 | 189 | 165 | 121 |
| | | | | | | | | | | | | signal intensity VEGF-A/GAPDH | | | | | |
| S.W. | 34 | pre | 5.5 | 39 | 0.1 | Ductal carcinoma | II–III | 6.0 | + | ++ | +++ | 0.290 | 0.568 | 0.764 | 0.116 | 0.282 | 0.708 |
| U.K. | 35 | pre | 4.9 | 43 | 2.4 | Ductal carcinoma | II–III | 1.7 | + | +++ | ++ | 0.200 | 0.767 | 1.029 | 0.172 | 0.709 | 0.957 |
| I.S. | 36 | pre | 13.0 | 15 | 0.1 | Mikroinv. duct. carc. | – | 1.5 | – | n.d. | n.d. | 0.229 | 0.555 | 1.194 | 0.281 | 0.513 | 1.018 |
| E.F. | 40 | pre | 3.6 | 59 | 9.8 | Intraduct. ca. in situ | I–II | 1.8 | – | ++ | +++ | 0.000 | 0.070 | 0.159 | 0.464 | 1.120 | 1.452 |
| A.W. | 41 | pre | 6.6 | 48 | 1.9 | Ductal carcinoma | II–III | 4.0 | + | 0 | 0 | 0.157 | 0.378 | 0.697 | 0.362 | 0.713 | 0.816 |
| I.Gr. | 42 | pre | 8.0 | 128 | 0.2 | Ductal carcinoma | II | 1.5 | – | 0 | 0 | 0.224 | 0.561 | 0.818 | 0.000 | 0.049 | 0.217 |
| S.K. | 42 | pre | 12.0 | 21 | 0.1 | Lobular carcinoma | II | 1.3 | + | +++ | ++ | 0.277 | 0.423 | 0.586 | 0.128 | 0.331 | 0.668 |
| A.B. | 46 | pre | ND | ND | ND | Lobular carcinoma | II | 1.8 | – | ++ | +++ | 0.000 | 0.832 | 1.158 | 0.000 | 0.136 | 0.216 |
| E.C. | 48 | pre | ND | ND | ND | Ductal carcinoma | II–III | 8.0 | + | + | 0 | 0.201 | 0.582 | 0.821 | 0.084 | 0.518 | 0.598 |
| C.Ma. | 50 | pre | 6.5 | 62 | 11 | Ductal carcinoma | II–III | 1.8 | + | +++ | +++ | 0.122 | 0.224 | 0.508 | 0.043 | 0.183 | 0.639 |
| I.Gf. | 56 | pre | 7.6 | 57 | 0.1 | Ductal carcinoma | II | 1.0 | – | ++ | ++ | 0.000 | 0.501 | 1.002 | 0.300 | 0.471 | 0.754 |
| H.P. | 50 | post | 42 | 44 | 0.1 | Ductal carcinoma | II | 2.2 | – | 0 | 0 | 0.127 | 0.181 | 0.467 | 0.049 | 0.108 | 0.499 |
| C.Me. | 52 | post | ND | ND | ND | Ductal carcinoma | II–III | 5.5 | + | + | 0 | 0.000 | 0.311 | 0.329 | 0.218 | 0.604 | 0.851 |
| E.S. | 60 | post | 30 | ND | ND | Duct. & lobul. carc. | II | 7.0 | + | ++ | ++ | 0.114 | 0.591 | 0.866 | 0.145 | 0.691 | 0.810 |
| E.R. | 67 | post | 98 | 4 | 0.1 | Ductal carcinoma | II | 4.0 | + | ++ | + | 0.101 | 0.116 | 0.358 | 0.220 | 0.858 | 0.847 |
| M.B. | 71 | post | 26 | 16 | 0.1 | Ductal carcinoma | II | 1.7 | + | ++ | ++ | 0.000 | 0.066 | 0.183 | 0.238 | 0.596 | 0.924 |
| M.M. | 74 | post | 52 | 15 | 0.1 | Labular carcinoma | II–III | 3.5 | – | ++ | ++ | 0.076 | 0.108 | 0.274 | 0.130 | 0.666 | 0.808 |
| R.B. | 81 | post* | 100 | 56 | 0.2 | Ductal carcinoma | II | 2.2 | – | ++ | ++ | 0.165 | 0.654 | 0.918 | 0.126 | 0.796 | 0.931 |
| E.B. | 82 | post | ND | ND | ND | Ductal carcinoma | II | 2.5 | + | +++ | ++ | 0.075 | 0.149 | 0.220 | 0.166 | 0.618 | 0.927 |

MP: menopausal status; FSH: follicle stimulating hormone; E2: plasma estradiol levels; P4: plasma progesterone levels; *: oestrogen replacement therapy; TU: tumour; LN: lymph node; NNT: non-neoplastic tissue; Ca: carcinoma; ER: oestrogen receptor status (immunoreactive score 0 – +++); PR: progesterone receptor status (immunoreactive score 0 – +++); ND: not determined.

*© 1999 Cancer Research Campaign*

certain breast cancer cells (Nakamura et al, 1996; Hyder et al, 1998). Here, we report, to our knowledge for the first time, that in vivo VEGF-A-expression levels in non-neoplastic breast tissue decrease after menopause.

### MATERIALS AND METHODS

### Human breast tissue samples

Paired malignant and adjacent non-neoplastic breast tissue samples from 19 unselected women with primary breast cancer were included in the study. The study was approved by the institutional review board. The endocrine status was determined based upon menstrual history, history of previous steroid hormone treatments and determination of plasma levels of FSH, oestradiol and progesterone on the day before surgery. Postmenopausal status was defined by one of the following:

- no spontaneous menses for at least 1 year in women older than 55
- plasma FSH levels higher than 40 IU $l^{-1}$
- plasma oestradiol levels below 15 pg $ml^{-1}$.

Histology, grading, tumour size, node status and oestrogen- and progesterone receptor status are summarized in Table 1. Breast tissue containing the palpable tumour was put on ice immediately upon removal and a representative sample of approximately 5 × 5 × 5 mm was taken from the tumour, and from the macroscopically tumour-free margin of the surgical specimen at a distance of at least 2 cm. The paired samples were snap-frozen in liquid nitrogen within 15 min upon removal of the tissue and stored in a freezer at – 80°C for no longer than 6 months until mRNA-isolation.

### RNA-isolation

PolyA $^{+}$RNA (mRNA) was purified after binding to biotin-labelled oligo(dt)20 by extraction with streptavidin-coated magnetic beads using a commercially available kit (Boehringer, Mannheim, Germany), following the procedures outlined by the manufacturer. DNAse-treated samples were stored at –80°C.

### Quantitative RT-PCR-assay

Reverse transcription polymerase chain reaction (RT-PCR) kits were purchased from Perkin-Elmer (Überlingen, Germany). cDNAs were synthesized from 25–50 ng mRNA using random hexamer primers. cDNA synthesis was carried out as suggested by the kit protocol, except that the RNA was denatured at 70°C for 5 min before RT for 45 min at 42°C. After an incubation at 99°C for 5 min to inactivate the reverse transcriptase, 10 µl cDNA aliquots were used in subsequent PCR reactions. Primers for VEGF-A and glyceraldehyde-3-phosphate dehydrogenase (GAPDH) were designed based upon published human cDNA sequences sequences with NBI-Oligo primer analysis software (MedProbe, Oslo, Norway). Both sets of primers cross intron sequences of the respective genes allowing to control for contaminating genomic DNA which would be detected by amplification of additional larger bands. Negative controls without mRNA were also performed. Custom synthesized VEGF-A primers (forward: 5′-CTGCTGTCTTGGGTGCATTGG-3′, reverse: 5′-CACCGC-CTCGGCTTGTCACAT-3′) and GAPDH primers (forward: 5′-GGCACCGTCAAGGCTGAGA-3′, reverse: 5′-AGCAGAGGG-GGCAGAGATGAT-3′) were obtained from Biometra (Göttingen, Germany). Quantitative PCR for VEGF-A was carried out using GAPDH as an internal standard in the same tube similar as described previously (Greb et al, 1997). Briefly, 10 µl of cDNA were preamplified for 7 cycles (94°C 45 s, 72°C 120 s) with VEGF-A-primers by adding 70 µl mastermix (1.88 mM magnesium chloride, 50 mM potassium chloride, 10 mM Tris–HCl pH 8.3, 1.5 U Ampli*Taq* DNA Polymerase, 0.25 µg Taq Start Antibody, Clontech, Heidelberg, Germany, 1.125 µM VEGF-A-primer). In preliminary experiments the preamplification of cDNAs with VEGF-A primers for 7 cycles before adding GAPDH primers was found to result in equal amplification efficiencies of GAPDH and VEGF-A-isoforms in the range PCR products were detectable by polyacrylamide gel electrophoresis. Subsequently, 10 µl mastermix containing GAPDH-primers (1.125 µM) were added and amplification continued (94°C 45 s, 65°C 45 s, 72°C 134 s + 2 s extension per cycle) until aliquots of the reaction were removed every 3 cycles for kinetic analysis. The range of PCR-cycles, in which parallel amplification occurred for VEGF-A and GAPDH PCR-products, was determined for each cDNA sample in preliminary experiments. In subsequent experiments 20 µl aliquots of PCR-products were removed starting between 14 and 26 cycles after adding GAPDH-primer depending on the optimal amplification range of the sample. At least four aliquots were removed every 3 cycles for later kinetic analysis in all samples. PCR-products were subjected to 5% polyacrylamide gel electrophoreses and the intensity of ethidium bromide-stained bands was determined from Polaroid photographs by the Lumi-Imager Analysis System (Boehringer, Mannheim, Germany). In all samples, a kinetic analysis was performed by plotting the signal intensity of PCR-products against the number of PCR-cycles (Figure 1). Relative VEGF-A gene expression levels was expressed for each VEGF mRNA isoform as the ratio between the arbitrary signal intensity of the respective VEGF-A-PCR product divided by the signal intensity of the GAPDH-derived PCR product in the range where parallel amplification could be demonstrated in the kinetic analysis plot. The intra- and interassay coefficients of variation were 9% and 22% respectively.

### Southern hybridization

The identity of PCR-products was confirmed by Southern hybridization employing 30-mer oligonucleotide probes specific to the VEGF-A (located in exon 3 and exon 7 respectively,5′-GCCATCCTGTGTGCCCCTGATGCGATGCGG-3′, 5′ CCTGTG-GGCCTTGCTCAGAGCGGAGAAAGC-3′ TIB MolBiol, Berlin, Germany) and GAPDH cDNA sequences (5′-CCACCATGGAGAAGGCTGGGGCTCATTTGC-3′…TGGGC… Biometra, Göttingen, Germany) internal to the PCR-primers as described previously (Greb et al, 1997). The exon 3-specific probe is expected to hybridize with all VEGF-A isoforms, the exon 7-specific probe is expected to hybridize only with VEGF 189 and VEGF 165.

### Statistics

The data were analysed by the two-way-ANOVA, Student's *t*-test and regression analysis. Bars indicate mean ± standard error.

© 1999 Cancer Research Campaign

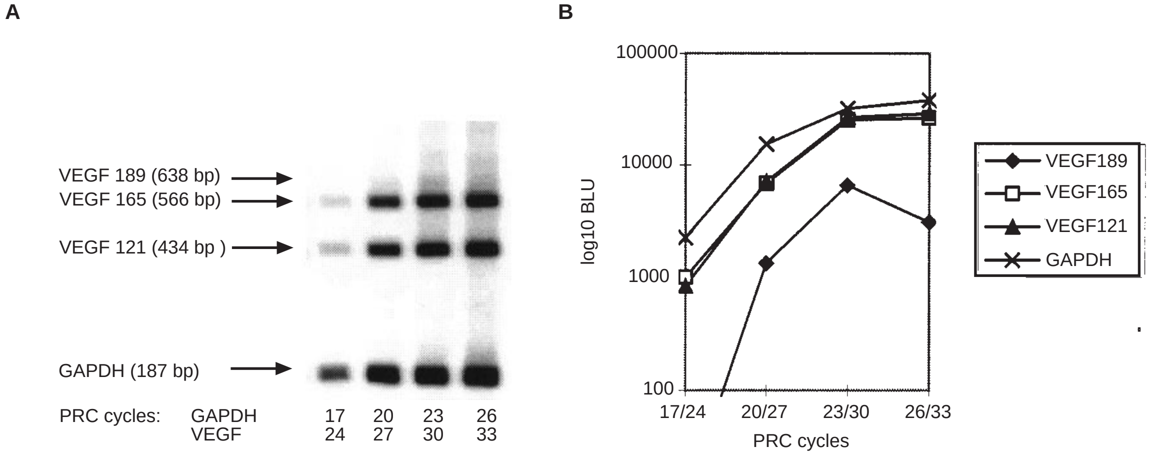

**Figure 1** A representative sample showing kinetic analysis of RT-PCR products. (**A**) After preamplification of cDNA with VEGF-A primers for seven PCR-cycles, GAPDH primers were added to the reaction. Aliquots of PCR products were removed from the reaction after 17, 20, 23 and 26 cycles and subjected to polyacrylamide gel electrophoresis. The intensity of the bands was determined from digitized images of polaroid photographs using the Lumi-Imager Analysis system in arbitrary Boehringer Light Units (BLU). (**B**) In all samples the ratio between VEGF-A isoforms and GAPDH was calculated from BLUs during the phase of parallel, exponential amplification. In this case the means of values obtained at 20/27 and at 23/30 cycles were used

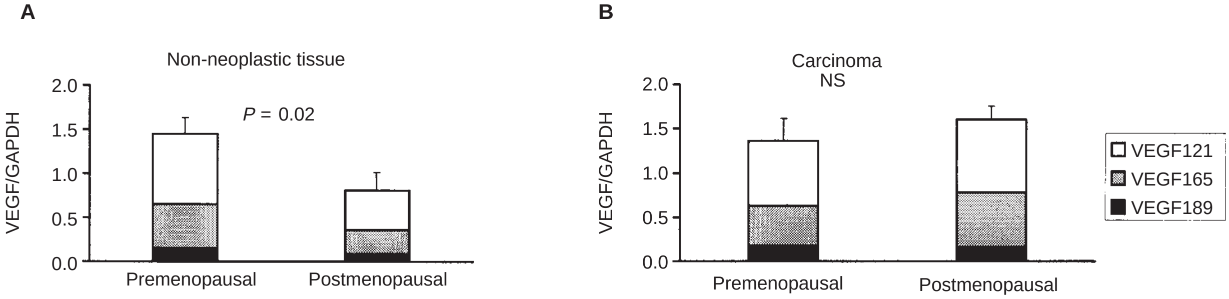

**Figure 2** Relative expression levels of VEGF-A isoforms. In non-neoplastic (**A**) breast tissue VEGF-A isoform expression was significantly higher in premenopausal women compared to post-menopausal women (means ± s.e.m.). No difference was found in tissues from cancer lesions (**B**)

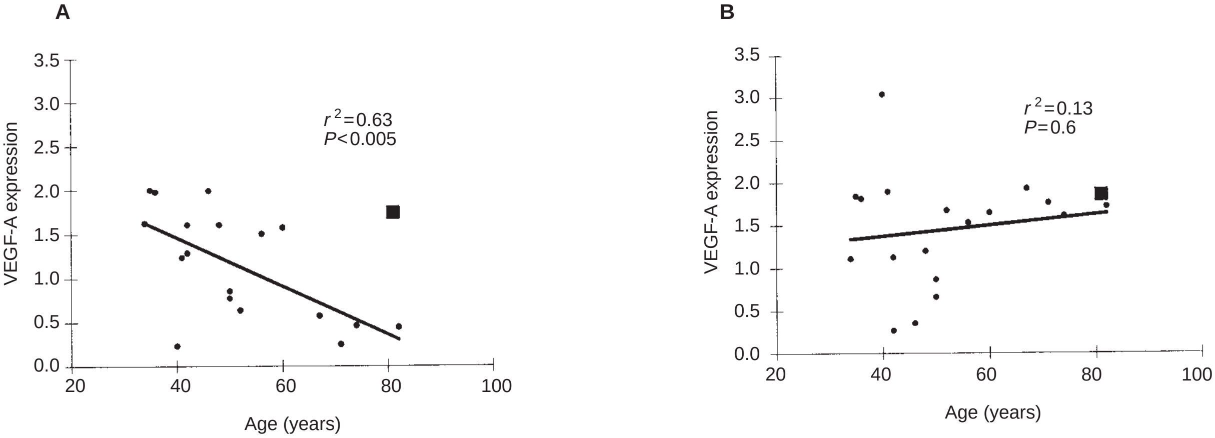

**Figure 3** Correlation of VEGF-A expression with age. In non-neoplastic tissue (**A**), patient age and VEGF-A-expression (sum of VEGF-A isoforms) were inversely correlated. No significant correlation could be demonstrated in cancerous tissue (**B**). In postmenopausal women, only one 81-year-old patient had received oestrogen replacement therapy for several years prior to surgery (black square (■), data point shown but not included in data analysis)

### RESULTS

RT-PCR was used to determine the relative expression levels of VEGF-A mRNAs coding for the isoforms VEGF189, VEGF165 and VEGF121, referenced to GAPDH expression in breast cancer lesions and the surrounding non-neoplastic tissue depending on the hormonal exposure in vivo (Figure 1). Clinical data and expression levels are summarized in Table 1. As shown in Figure 2, only in non-neoplastic tissue was VEGF-A expression dependent on the patient's menopausal status. In normal breast tissue,

© 1999 Cancer Research Campaign the expression of all detected VEGF-A isoforms was significantly higher in premenopausal compared to post-menopausal women ($P$ = 0.02). In contrast, VEGF-A expression levels in cancer lesions were similar between the two groups of patients. Consequently, in those patients not having received hormone replacement therapy prior to breast surgery, VEGF-A expression and age were inversely correlated in non-neoplastic breast tissue ($P$ < 0.006), but not in cancer lesions (Figure 3), indicating that an age-dependent decrease of steroidal exposure of the tissue might influence VEGF-A expression in breast tissue. The relative ratio of VEGF-A isoforms to each other was similar between benign (189/165/121 = 11%/34%/55%) and malignant (189/165/121 = 12%/36%/53%) tissues. Other clinical parameters, such as tumour size, presence or absence of lymph node metastases and oestrogen receptor/progesterone receptor-status were not significantly associated with VEGF-A expression levels in cancer lesions (Table 1).

PCR-product identity could be verified by sequence specific probe hybridization (Southern hybridization). Sequential probing with three specific oligonucleotides recognizing sequences situated in exon 3 and exon 7 of the human VEGF gene and a sequence of the human GAPDH gene respectively, gave the expected signal patterns. The GAPDH-specific probe resulted in a band at the position of the corresponding PCR product at 187 base pairs (bps), VEGF–exon 7-specific probe gave two bands at the location of VEGF189- and VEGF165-derived PCR products at 638 and 566 bps and VEGF–exon 3-specific probe gave three bands at the location of VEGF189-, VEGF165- and VEGF121-derived PCR products (638, 566 and 434 bps) (data not shown).

### DISCUSSION

To our knowledge, this is the first report that in normal breast tissue in vivo VEGF-mRNA expression levels decrease age-dependently, suggesting that the ovarian steroids might be involved in the regulation of this key angiogenic factor. However, differences in VEGF-A expression levels between pre- and post-menopausal patients are only detectable in normal breast tissue and not in the cancerous lesion itself. Based upon our data, the hormonal dependency of VEGF-A expression seems to disappear in the majority of cancerous cells in breast tumours which would be compatible with our understanding that onset and development of cancer appears to involve tumour desensitization to host regulatory signals (Dickson, 1992).

Interestingly, these preliminary findings are in line with previous studies assessing angiogenesis in breast tissue indirectly by perfusion measurements in vivo. Villena-Heinsen et al (1998) reported a significant correlation between Doppler sonographically measured vascular resistance indices and age in normal breast tissue of post-menopausal women. This phenomenon could not be demonstrated in premenopausal women or in tumour lesions. The influence of the ovarian hormones on vascular functions in the breast is also supported by MRI data of the human breast. Müller-Schimpfle et al (1997) reported parenchymal contrast enhancement patterns which fluctuated depending on the patients' age and stage of the menstrual cycle. The lowest enhancement was found in women younger than 35 and older than 50 years, and if the examination was performed between cycle days 7–20 compared to cycle days 21–26. The results of our study substantiate previous reports which failed to show an influence of menopausal status or age on cytosolic levels of VEGF-protein in breast cancer lesions (Gasparini et al, 1997). Also, the constant ratio of VEGF-A isoforms relative to each other (VEGF121 > VEGF 165 > VEGF 189) as observed by us in vivo, seems to reflect parallel changes of all isoforms, e.g. under different culture conditions of breast cancer cell lines. The highly soluble protein VEGF121 which is diffusable into the surrounding tissue seems to be the predominant isoform in breast cells (Scott et al, 1998*a*).

Total GAPDH-normalized VEGF-A mRNA expression in cancer lesions compared to the surrounding tissue appeared to be up-regulated only in post-menopausal patients in our study. The absence of VEGF-A up-regulation in breast carcinomas compared to matched normal breast tissue in our series of premenopausal patients seems to be contradictory to the dramatic up-regulation of VEGF in carcinomas compared to their non-neoplstic counterparts as described by others (Brown et al, 1995; Anan et al, 1996; Yoshiji et al, 1996; Obermair et al, 1997; Scott et al, 1998*b*). A possible explanation for the discrepancy of our data with other studies might be the use of GAPDH as a reference gene in the quantitative RT-PCR assay. GAPDH was demonstrated as one of several genes which are up-regulated in breast ductal carcinoma compared to histologically normal tissue (Bini et al, 1997) and a significant correlation of VEGF mRNA and GAPDH mRNA expression was found in breast carcinomas (Scott et al, 1998*b*), possibly because both genes are regulated by hypoxia (Graven et al, 1998). Potentially, an increased number of VEGF mRNA transcripts parallel to an up-regulation of GAPDH in carcinomatous tissue might have caused the similar VEGF/GAPDH ratios in malignant and benign breast tissue allowing only separate comparisons in our study for benign and malignant tissue (Figure 2). For this reason no conclusions can be drawn comparing benign and malignant tissue to each other. An up-regulation of VEGF expression in breast carcinomas compared to normal breast tissue using β-actin as a reference gene was demonstrated by Northern blotting (Yoshiji et al, 1996) and RT-PCR detectability (Anan et al, 1996). Similar to our results, Scott et al (1998*b*) measured insignificantly higher VEGF mRNA expression levels in the tumour compared to the surrounding normal tissue by ribonuclease protection assay using an external spiked control. The range of VEGF expression between these two groups overlapped to some extend. Subjective evaluation of tissue sections by in situ hybridization suggested stronger staining in malignant breast epithelial cells (Brown et al, 1995), but a significant amount of signal for VEGF mRNA could also be observed in normal breast epithelial cells (Scott et al, 1998*b*). Taking these studies together, there is strong evidence that VEGF mRNA expression is indeed up-regulated in breast cancer tissue compared to normal breast which fits to measurements of VEGF protein production in breast cancer lesions and matched control samples (Obermair et al, 1997). Despite possible difficulties in comparing relative VEGF mRNA expression levels between malignant and benign tissues our findings in each type of tissue with respect to age and hormone dependency appear nevertheless to be valid because GAPDH gene expression is unlikely influenced by the hormonal milieu. GAPDH mRNA has been shown to be broadly expressed across breast tissue sections (Wright et al, 1997) and not to be regulated by hormonal steroids in breast cells (Le-Bihan et al, 1998) and human tumour xenografts (Wright et al, 1995). The validity of the quantitative measurements was carefully monitored in our study by controlling for amplification efficiencies of different RT-PCR products in all samples.

The dependency of VEGF-A mRNA expression in the breast from the endocrine milieu, as suggested by our study, extends previous findings addressing the regulation of VEGF-A in breast

© 1999 Cancer Research Campaign cells. Hypoxia was identified as the most potent stimulus of VEGF mRNA expression in several breast cancer cell lines (Scott et al, 1998*a*) and in normal mammary stromal cells (Hlatky et al, 1994) in agreement with the VEGF-mediated neovascularization of hypoxic areas near necrotic regions in human breast tumours (Wright et al, 1997).

Additionally, tumour angiogenesis, e.g. via up-regulation of VEGF expression, is thought to be the consequence of a multistep process caused by a sequence of genetic alterations in tumour cells. Mutations of the tumour suppressor gene p53 and over-expression of oncogenes like v-Src and ras were reported to be associated with increased expression of VEGF (Mukhopadhyay et al, 1995; Rak et al, 1996). Based upon our results, and the few reports on hormonal regulation of VEGF in breast cancer it cannot be ruled out that oestrogens and progestins may stimulate VEGF production in some breast cancer cells. Hyder et al (1998) demonstrated a progesterone-dependent up-regulation of VEGF in progesterone receptor-rich T47-D breast cancer cells but not in all other cell lines tested. This is in agreement with the observations of Scott et al (1998*a*) who failed to detect an effect of female reproductive hormones on VEGF mRNA expression in several breast cancer cell lines which did not include T47-D cells. In vivo, a hormonal dependency of VEGF could be demonstrated so far only in rat mammary tumours in which VEGF expression was shown to be stimulated by oestradiol (Nakamura et al, 1996).

In summary, the data of the present study support our hypothesis that the VEGF-mediated regulation of microvascular functions and angiogenesis in breast tissue is subject to ovarian steroid action. Since VEGF-A has emerged as an important prognostic parameter which closely correlates with microvascular density in breast cancer (Toi et al, 1995), our study raises several questions concerning the consequences of hormonal modulation of angiogenesis in the human breast. While the effects of endocrine treatments like anti-oestrogens and progestins on cell proliferation and tumour growth were studied extensively, we at present do not know how the angiogenic potential in breast cancer, in the surrounding tissue and in distant micrometastases is influenced by these treatments. Whether or not beneficial effects of endocrine therapies on growth are supported or counteracted by modulation of angiogenesis remains to be determined. Also, it is not yet clear whether the regulation of a key angiogenic factor by ovarian hormones in normal breast tissue plays a role in the aetiology of the disease and whether exogenously administered hormones like oral contraceptives and hormone replacement therapies are able to contribute to biologically relevant changes in the breast. The small group of patients investigated so far does not allow to test for a menstrual cycle-dependent effect on VEGF-A expression. However, those data might influence the controversially discussed issue of timing of breast cancer surgery during the menstrual cycle (Hagen and Hrushesky, 1998) and VEGF secretion of the breast could contribute to menstrual cycle-dependent changes in serum levels of VEGF (Benoy et al, 1998; Chung et al, 1998; Heer et al, 1998). Since MRI of the breast seems to be especially susceptible for VEGF-induced changes in microvascular functions like vessel permeability, as could be shown by anti-VEGF treatment in nude mice (Pham et al, 1998), it seems reasonable to expect that the hormonal exposure of the breast influences parenchymal contrast enhancement patterns. This may explain why these patterns are changing depending on the stage of the patients' menstrual cycle (Müller-Schimpfle et al, 1997).

In conclusion, our observation that VEGF-A mRNA-expression levels in normal breast tissue are higher in premenopausal than post-menopausal breast cancer patients raises further questions about the effects of endogenous and therapeutic hormonal steroids on the angiogenic phenotype of the human breast and its effects on breast cancer.

### ACKNOWLEDGEMENTS

We acknowledge gratefully the assistance with sampling fresh tissue of Dr M Geppert, Ms H Böhner, Ms G Haug and Ms G Wroblewski of our histology laboratory.

### REFERENCES

Anan K, Morisaki T, Katano M, Ikubo A, Kitsuki H, Uchiyama A, Kuroki S, Tanaka M and Torisu M (1996) Vascular endothelial growth factor and platelet-derived growth factor are potential angiogenic and metastatic factors in human breast cancer. *Surgery* **119**: 333–339

Beatson GT (1896) On the treatment of inoperable cases of carcinoma of the mamma: suggestions for a new method of treatment with illustrative cases. *Lancet* **ii**: 104–107

Benoy I, Vermeulen P, Wuyts H and Dirix L (1998) Vascular endothelial cell growth factor (VEGF) serum concentrations change according to the phase of the menstrual cycle. *Eur J Cancer* **34**: 1298–1299

Bini L, Magi B, Marzocchi B, Arcuri F, Tripodi S, Cintorino M, Sanchez JC, Frutiger S, Hughes G, Pallini V, Hochstrasser DF and Tosi P (1997) Protein expression profiles in human breast ductal carcinoma and histologically normal tissue. *Electrophoresis* **18**: 2832–2841

Brown LF, Berse B, Jackman RW, Tognazzi K, Guidi AJ, Dvorak HF, Senger DR, Connolly JL and Schnitt SJ (1995) Expression of vascular permeability factor (vascular endothelial growth factor) and its receptors in breast cancer. *Hum Pathol* **26**: 86–91

Buadu LD, Murakami J, Murayama S, Hashiguchi N, Sakai S, Masuda K, Toyoshima S, Kuroki S and Ohno S (1996) Breast lesions: correlation of contrast medium enhancement patterns on MR images with histopathologic findings and tumor angiogenesis. *Radiology* **200**: 639–649

Chung HC, Rha SY, Ahn JB, Shim KY, Yoo NC, Kim JH, Roh JK, Lee KS, Min JS, Kim BS and Kim JJ (1998) Menstrual state should be considered in determining sero-positivity of soluble angiogenic factors in breast cancer. *Int J Mol Med* **2**: 465–470

Dickson RB (1992) Regulation of tumor–host interactions in breast cancer. *J Steroid Biochem Mol Biol* **41**: 389–400

Ferrara N and Davis-Smyth T (1997) The biology of vascular endothelial growth factor. *Endocr Rev* **18**: 4–25

Gasparini G, Toi M, Gion M, Verderio P, Dittadi R, Hanatani M, Matsubara I, Vinante O, Bonoldi E, Boracchi P, Gatti C, Suzuki H and Tominaga T (1997) Prognostic significance of vascular endothelial growth factor protein in node negative breast carcinoma. *J Natl Cancer Inst* **89**: 139–147

Gimbrone MA Jr, Leapman SB, Cotran RS and Folkman J (1972) Tumor dormancy in vivo by prevention of neovascularization. *J Exp Med* **136**: 261–276

Graven KK, McDonald RJ and Farber HW (1998) Hypoxic regulation of endothelial glyceraldehyde-3-phosphate dehydrogenase. *Am J Physiol* **274**: C347–355

Greb RR, Heikinheimo O, Williams RF, Hodgen GD and Goodman AL (1997) Vascular endothelial growth factor in primate endometrium is regulated by oestrogen receptor and progesterone receptor ligands in vivo. *Hum Reprod* **12**: 1280–1292

Hagen AA and Hrushesky WJM (1998) Menstrual timing of breast cancer surgery. *Am J Surg* **175**: 245–261

Hanahan D and Folkman J (1996) Patterns and emerging mechanisms of the angiogenic switch during tumorigenesis. *Cell* **86**: 353–364

Heer K, Kumar H, Speirs V, Greenman J, Drew PJ, Fox JN, Carleton PJ, Monson JRT and Kerin MJ (1998) Vascular endothelial growth factor in premenopausal women: indicator of the best time for breast cancer surgery? *Br J Cancer* **78**: 1203–1207

Hlatky L, Tsionou C, Hahnfeldt P and Coleman CN (1994) Mammary fibroblasts may influence breast tumor angiogenesis via hypoxia-induced vascular endothelial growth factor up-regulation and protein expression. *Cancer Res* **54**: 6083–6086

 © 1999 Cancer Research Campaign

Hyder SM, Murthy L and Stancel GM (1998) Progestin regulation of vascular endothelial growth factor in human breast cancer cells. *Cancer Res* **58**: 392–395

Le-Bihan S, Marsaud V, Mercier-Bodard C, Baulieu EE, Mader S, White JH and Renoir JM (1998) Calcium/calmodulin kinase inhibitors and immunosuppressant macrolides rapamycin and FK506 inhibit progestin- and glucocorticosteroid receptor-mediated transcription in human breast cancer T47D cells. *Mol Endocrinol* **12**: 986–1001

Müller-Schimpfle M, Ohmenhäuser K, Stoll P, Dietz K and Claussen CD (1997) Menstrual cycle and age: influence on parenchymal contrast medium enhancement in MR imaging of the breast. *Radiology* **203**: 145–149

Mukhopadhyay D, Tsiokas L and Sukhatme VP (1995) Wild-type p53 and v-src exert opposing influences on human vascular endothelial growth factor gene expression. *Cancer Res* **55**: 6161–6165

Nakamura J, Savinov A, Lu Q and Brodie A (1996) Estrogen regulates vascular endothelial growth permeability factor expression in 7,12-dimethylbenz(a)anthracene-induced rat mammary tumors. *Endocrinology* **137**: 5589–5596

Obermair A, Kucera E, Mayerhofer K, Speiser P, Seifert M, Czerwenka K, Kaider A, Leodolter S, Kainz C and Zeillinger R (1997) Vascular endothelial growth factor (VEGF) in human breast cancer: correlation with disease free survival. *Int J Cancer* **74**: 455–458

Pham CD, Roberts TPL, vanBruggen N, Melnyk O, Mann J, Ferrara N, Cohen RL and Brasch RC (1998) Magnetic resonance imaging detects suppression of tumor vascular permeability after administration of antibody to vascular endothelial growth factor. *Cancer Invest* **16**: 225–230

Rak J, Filmus J and Kerbel RS (1996) Reciprocal paracrine interactions between tumour cells and endothelial cells: the angiogenesis progression hypothesis. *Eur J Cancer* **32A**: 2438–2450

Scott PAE, Gleadle JM, Bicknell R and Harris AL (1998*a*) Role of the hypoxia sensing system, acidity and reproductive hormones in the variability of vascular endothelial growth factor induction in human breast carcinoma cell lines. *Intern J Cancer* **75**: 706–712

Scott PAE, Smith K, Poulsom R, Debenedetti A, Bicknell R and Harris AL (1998*b*) Differential expression of vascular endothelial growth factor MRNA vs protein isoform expression in human breast cancer and relationship to eIF-4E. *Br J Cancer* **77**: 2120–2128

Toi M, Inada K, Suzuki H and Tominaga T (1995) Tumor angiogenesis in breast cancer: its importance as a prognostic indicator and the association with vascular endothelial growth factor expression. *Breast Cancer Res Treat* **36**: 193–204

Villena-Heinsen C, Ertan AK, Hollander M, Konig J, Tossounidis I and Schmidt W (1998) Diagnostic and predictive ranking of the vascular resistance index in breast tumours. *Ultraschall in der Medizin* **19**: 10–15

Vogel PM, Georgiade NG, Fetter BF, Vogel FS and McCarty KS Jr (1981) The correlation of histologic changes in the human breast with the menstrual cycle. *Am J Pathol* **104**: 23–34

Wright PS, Cross-Doersen DE, Chmielewski PA, Bush TL, Bitonti AJ and Miller JA (1995) Measurement of mRNA levels in tumor xenografts with quantitative autoradiography and in situ hybridization. *FASEB J* **9**: 279–283

Wright PS, Loudy DE, Cross-Doersen DE, Montgomery LR, Sprinkle-Cavallo J, Miller JA, Distler CM, Lower EE and Woessner RD (1997) Quantitation of vascular endothelial growth factor mRNA levels in human breast tumors and metastatic lymph nodes. *Exp Mol Pathol* **64**: 41–51

Yoshiji H, Gomez DE, Shibuya M and Thorgeirsson UP (1996) Expression of vascular endothelial growth factor, its receptor, and other angiogenic factors in human breast cancer. *Cancer Res* **56**: 2013–2016

*© 1999 Cancer Research Campaign*
